# Folate-targeted star-shaped cationic copolymer co-delivering docetaxel and MMP-9 siRNA for nasopharyngeal carcinoma therapy

**DOI:** 10.18632/oncotarget.9771

**Published:** 2016-06-01

**Authors:** Tao Liu, Xidong Wu, Yigang Wang, Tao Zhang, Ting Wu, Fang Liu, Wansong Wang, Gang Jiang, Minqiang Xie

**Affiliations:** ^1^ Department of Otolaryngology, Zhujiang Hospital, Southern Medical University, Guangzhou, 510282, China; ^2^ Department of Pharmacology, Jiangxi Institute of Materia Medica, Nanchang, 330029, China; ^3^ School of Life Sciences, Zhejiang Sci-Tech University, Hangzhou, 310018, China; ^4^ Department of Light Chemical Engineering, Guangdong Polytechnic, Foshan, 528041, China; ^5^ Medical College of Nanchang University, Nanchang, 330038, China

**Keywords:** target, star-shaped, co-delivery

## Abstract

The co-delivery of drug and gene has become the primary strategy in cancer therapy. Based on our previous work, to co-deliver docetaxel (DOC) and MMP-9 siRNA more efficiently for HNE-1 nasopharyngeal carcinoma therapy, a folate-modified star-shaped copolymer (FA-CD-PLLD) consisting of β-cyclodextrin (CD) and poly(L-lysine) dendron (PLLD) was synthesized, and then used for DOC and MMP-9 co-delivery. Different from commonly used amphiphilic copolymers micelles, the obtained CD derivative could be used directly for the combinatorial delivery of nucleic acid and hydrophobic DOC without a complicated micellization process. *In vitro* and *in vivo* assays are carried out to confirm the effectiveness of the target strategy and combined treatment. It was found that the conjugation of CD-PLLD with FA could enhance the DOC/MMP-9 delivery effect obviously, inducing a more significant apoptosis and decreasing invasive capacity of HEN-1 cells. *In vivo* assays showed that FA-CD-PLLD/DOC/MMP-9 could inhibit HNE-1 tumor growth and decrease PCNA expression effectively, indicating a promising strategy for nasopharyngeal carcinoma therapy. Moreover, the *in vivo* distribution of DOC and MMP-9, blood compatibility and toxicity are also explored.

## INTRODUCTION

Nasopharyngeal carcinoma (NPC) is one of the most common malignant tumors in Southern China [1]. The comprehensive treatment of radiotherapy combined with chemotherapy is the main clinical treatment strategy for NPC nowadays. However, the average 5-year survival rate of advanced NPC patients is still low due to the easy tumor metastasis, multi-drug resistance and so on [2].

Recently, the strategy of combining chemotherapy with gene therapy has been promising in cancer therapy, because this technique could promote synergistic actions, enhance the treatment effect and deter the development of drug resistance [3]. For examples, He et al. prepared a drug and gene co-delivery system through coordination using Zn^2+^ as the connecting point. The obtained coordination liposome promoted the cellular uptake of cisplatin and siRNA, and enabled efficient endosomal escape in cisplatin-resistant ovarian cancer cells [4]. Li et al. used poly(carboxybetaine) to conjugate camptothecin, then assembled with cationic liposomes to form the drug and gene dual carrier, which showed a synergistic tumor suppression effect in tumor-bearing mice *in vivo* [5]. Chang et al. prepared the drug and gene co-delivering liposomes assembling from amphiphilic pillar [5] arene capped with ferrocenium, which showed redox sensitivity and effective drug/siRNA co-delivery [6].

In these assemble carriers, hydrophobic anticancer drugs were incorporated into the hydrophobic cores, and plasmid DNA or siRNA was bound to the hydrophilic shells with cationic character. For the preparation of these micelles, however, these self-assembly processes are usually difficult to control, which was not easy to obtain the stable and uniform complexes. Moreover, micelles was not stable in blood circulation *in vivo*, the disassembly of micelles may result in the drug emission [7–9].

Our collaborators have synthesized a star-shaped cyclodextrin derivative (CD-PLLD) consisting of a cyclodextrin (CD) core and poly(L-lysine) dendron (PLLD) arms [10]. The CD core could interact with hydrophobic model drug and the cationic arms could bind pEGFP respectively, in which a complicated micellization process is avoided. Moreover, PLLD is low cytotoxicity and high transfection efficiency, which has been applied widely in drug and gene delivery [11–14]. We have used this CD-PLLD to co-deliver docetaxel and MMP-9 siRNA plasmid for NPC therapy. *In vitro* assay indicated that CD-PLLD could co-deliver docetaxel (DOC) and MMP-9 effectively into NPC HNE-1 cells and the HNE-1 cells apoptosised obviously, which suggested a potential application in drug and gene co-delivery [15]. However, the deliver efficiency of CD-PLLD should be improved.

It is reported that folate receptors (FRs) are expressed at high levels in numerous cancers including NPC HNE-1 cells, while low expression of FRs was found in normal tissues [16–18]. Due to the strong affinity between FRs and folate, many works have been reported in which folate was used to modify carrier to facilitate the internalization of the carriers into cancer cells [19–22].

In this paper, we conjugated CD-PLLD with FA to synthesize a FA-targeted drug and gene dual carrier, and then use this carrier to co-deliver DOC and MMP-9 for NPC treatment. *In vitro* and *in vivo* assays are carried out to confirm the effectiveness of the target strategy and combined treatment. And the *in vivo* distribution of DOC and MMP-9 is also explored.

## RESULTS

### Synthesis of FA-CD-PLLD

**Scheme 1:**
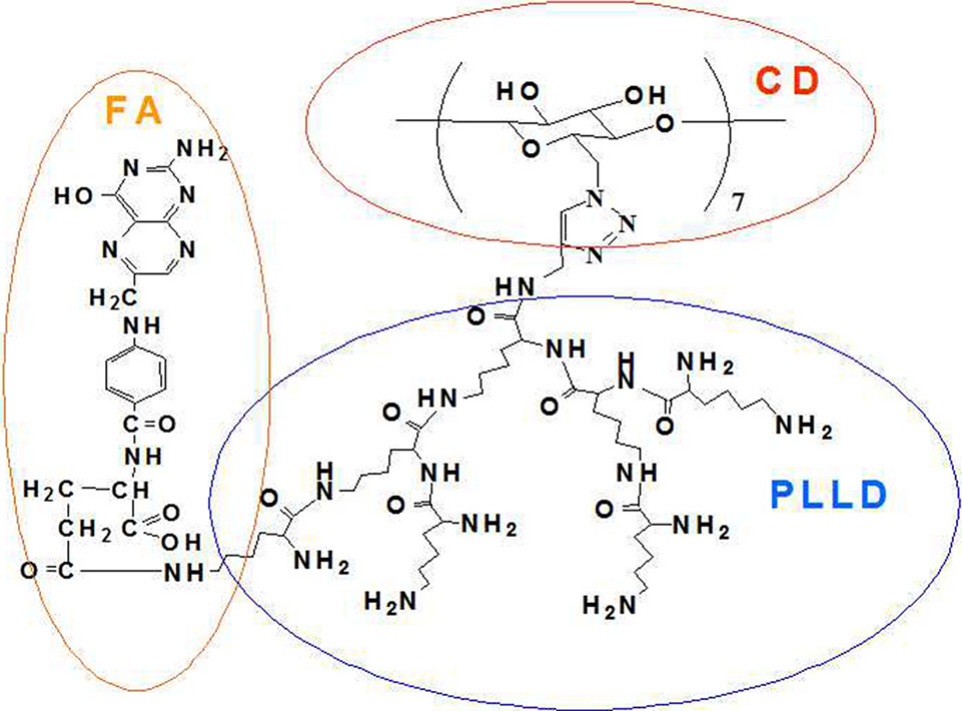
Chemical structure of FA-CD-PLLD

**Figure 1:**
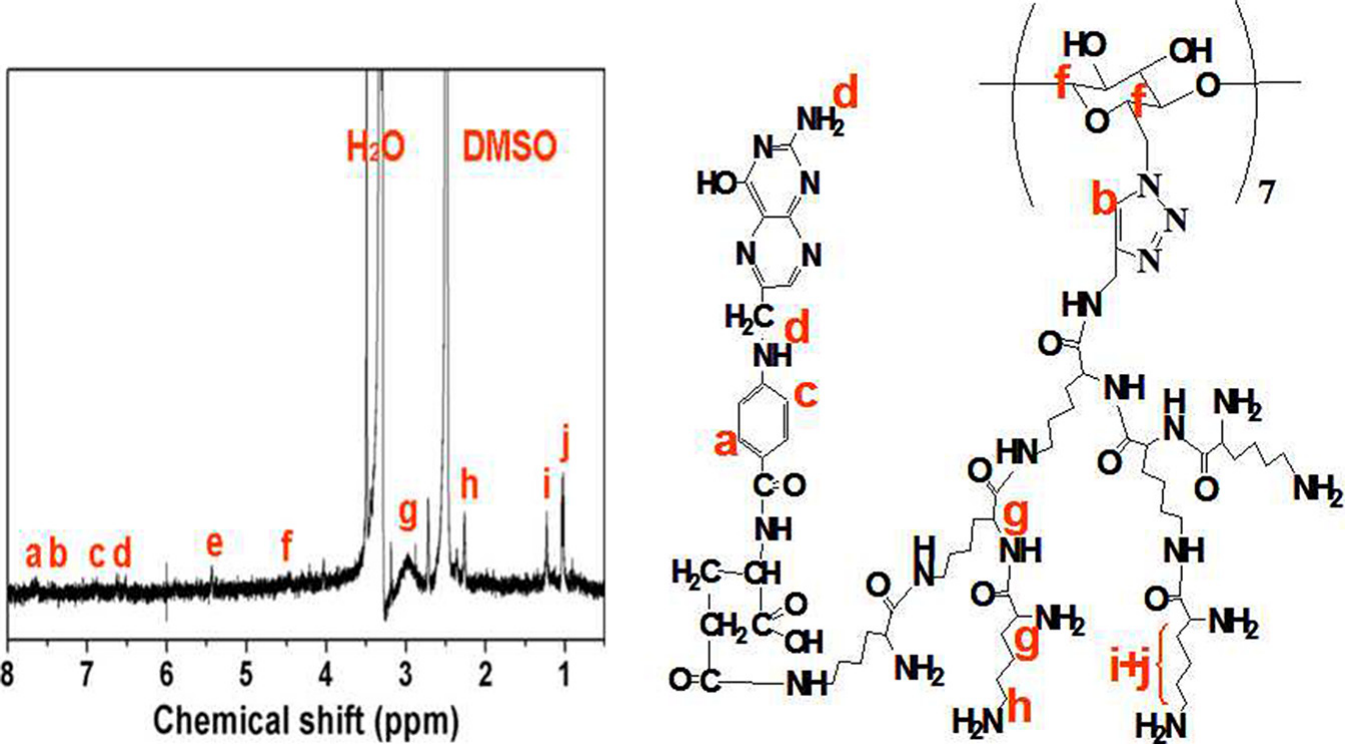
^1^H NMR spectra of FA-CD-PLLD (DMSO-d_6_; 25°C)

The star-shaped polymer, CD-PLLD, has been synthesized in our previous work and showed drug/gene co-delivery ability [15]. To improve the delivery ability of CD-PLLD to HNE-1 cells, FA was conjugated to CD-PLLD, and its chemical structure was shown in Scheme 1. FA was conjugated by the amidation reaction between the carboxyl groups of FA and amino groups of PLLD. ^1^H NMR was used to confirm this. From Figure 1, all signals of FA-CD-PLLD were marked. Except the peaks from CD-PLLD, the characterized peaks at 6.5–8.0 ppm were attributed to FA. This result indicated that the FA-functionalized CD-PLLD was obtained successfully. Particularly, the integral ratio (I_a_/I_b_ of the proton resonance signal at 7.65 ppm to that at 7.50 ppm was found to be about 2.58, which indicated that 1 mol of CD-PLLD reacted with about 2.58 mol of FA.

### *In vitro* and *in vivo* target

### *In vitro* target

**Figure 2:**
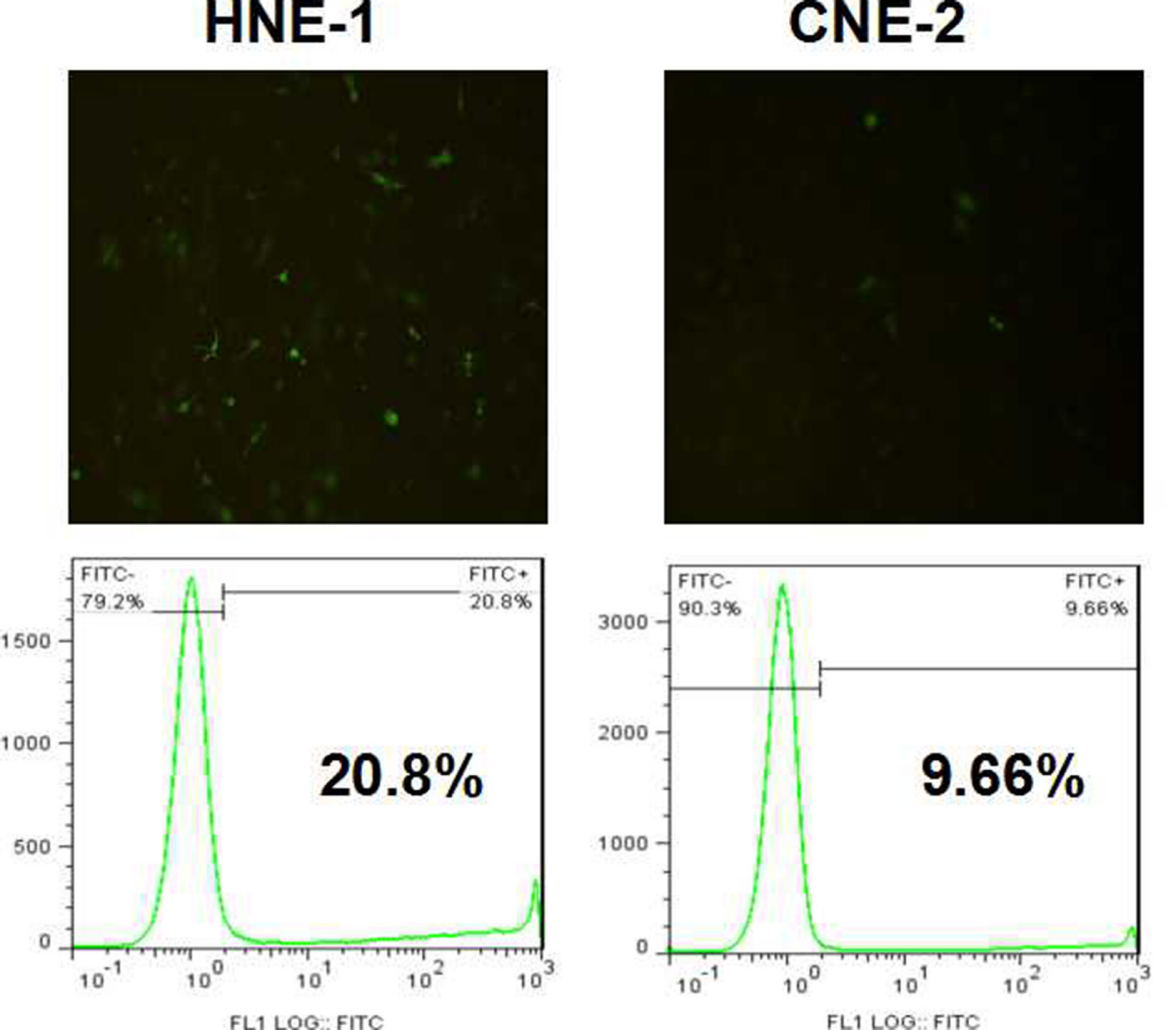
Transfection results of FA-CD-PLLD/MMP-9 into HNE-1 or CNE-2 cells

We evaluated the targeted transfection efficiency of MMP-9 siRNA plasmid into NPC cells. As shown in Figure 2, the gene transfection efficiency in CNE-2 cells with negative FR expression was lower, and less than 10% CNE-2 cells were transfected. Correspondingly, the HNE-1 cells with high FR expression showed significantly higher transfection efficiency than that in CNE-2 cells, and more than 2 times of HNE-1 cells were transfected. The reason was resulted from the molecular targeting phagocytosis effect in addition to nonspecific phagocytosis effect.

**Figure 3:**
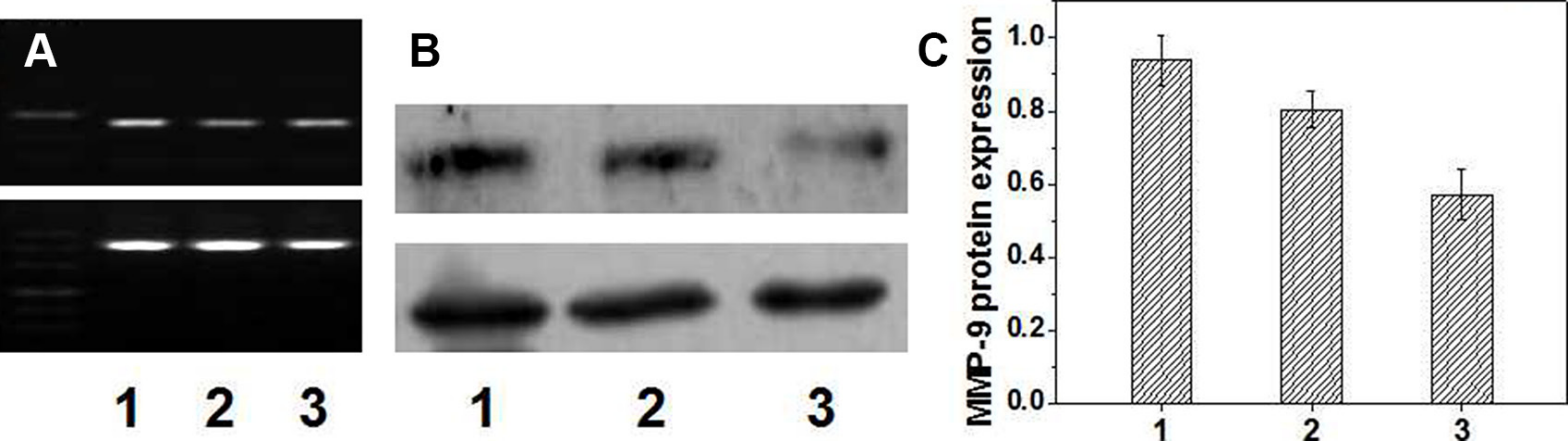
PCR (A) and western-blot (B and C) results (*n* = 5) (1: FA-CD-PLLD; 2: CD-PLLD/MMP-9; 3: FA-CD-PLLD/MMP-9).

To study further the targeting transfection effect of FA-CD-PLLD/MMP-9 complexes to HNE-1 cells, RT-PCR and Western blot analysis was carried out to detect MMP-9 mRNA and protein expression. From the result of Figure 3A, it was found that after targeting sequence-specific MMP-9 gene silencing by FA-CD-PLLD/MMP-9 complexes, the MMP-9 mRNA expression level was reduced obviously. Its MMP-9 mRNA expression reduced about 20% compared with CD-PLLD/MMP-9 group, although CD-PLLD/MMP-9 mediated also the reduction of MMP-9 mRNA. A reduction in MMP-9 mRNA was subsequently accompanied by decreased MMP-9 protein expression (Figure 3B and 3C), as determined by Western blot analyses of MMP-9 protein in the cell lysates after targeting transfection. It was found the samples treated with targeting FA-CD-PLLD/MMP-9 showed the obvious reduction of MMP-9 protein expression compared to the non-targeting CD-PLLD/MMP-9, and the MMP-9 protein expression reduced about 25%.

### *In vivo* target

**Figure 4:**
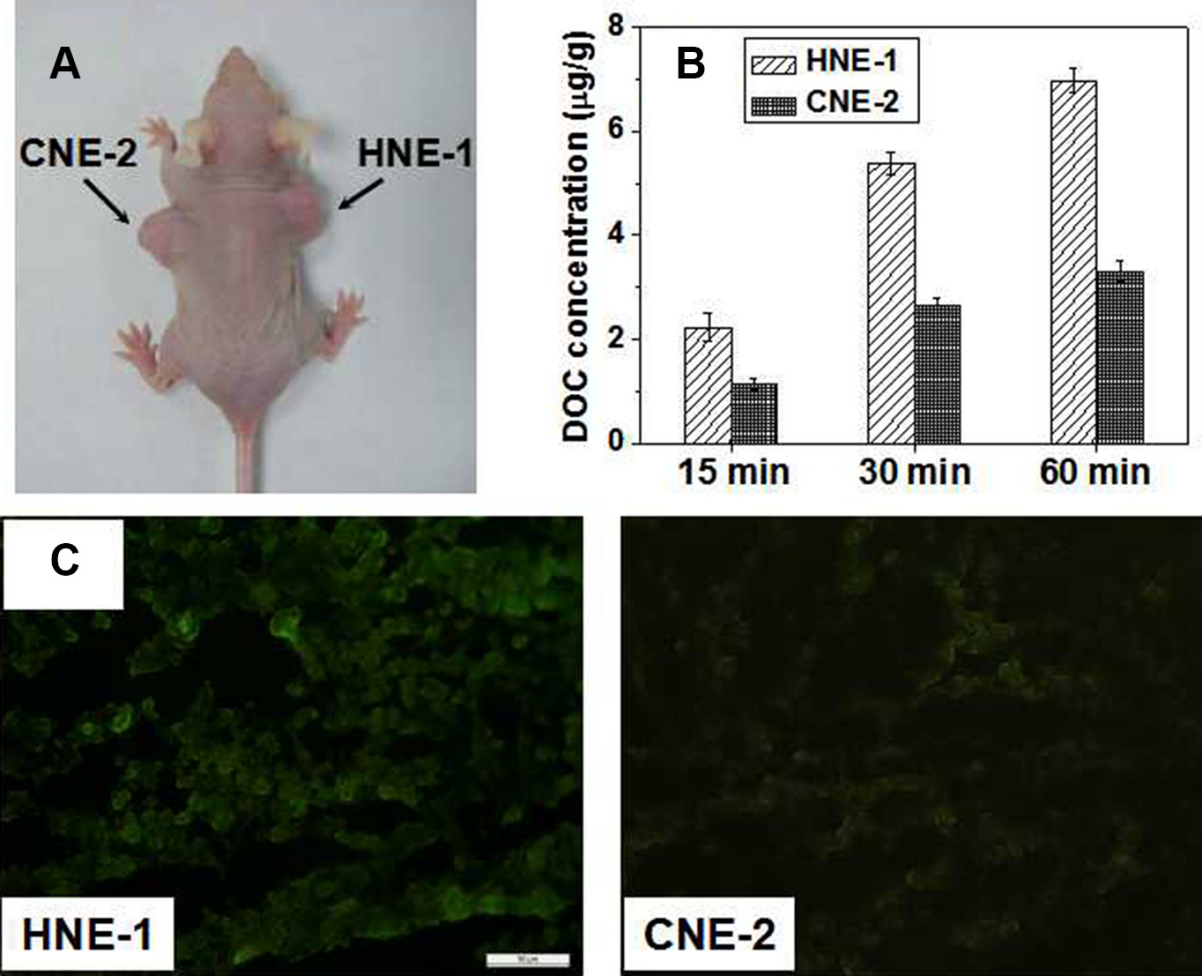
(**A**) A representative mice co-bearing HNE-1 and CNE-2 tumors. (**B**) DOC concentrations in HNE-1 and CNE-2 tumor after different injection time (*n* = 5). (**C**) GFP expression in HNE-1 and CNE-2 tumor.

To confirm the targeting delivery of FA-CD-PLLD/DOC/MMP-9, the nude mice bearing both HNE-1 and CNE-2 tumor model were set as shown in Figure 4A.

It showed a good linear relationship between DOC concentration in tumor tissues and peak area, and the standard curve of DOC and regression equation were achieved as follow:

In HNE-1 tumor: Y (peak area) = 2474.3 × (DOC concentration) + 799.96;

In CNE-2 tumor: Y (peak area) = 2496.8 × (DOC concentration) − 272.89.

Then, the DOC concentration in tumor tissue at different time (5 min, 30 min and 60 min) after tail intravenous injection with FA-CD-PLLD/DOC/MMP-9 were calculated, average concentrations of DOC in both HNE-1 and CNE-2 tumor was 2.33 and 1.16 μg/g at 15 min, 5.39 and 2.65 μg/g at 30 min, 6.97 and 3.30 μg/g at 60 min respectively, and the results were shown in Figure 4B.

Via tail intravenous injection with nanocomposite for 24 h, GFP expression in tumor tissues were shown in Figure 4C. A stronger GFP expression was observed in HNE-1 tumor than in CNE-2 tumor, which was consistent with the DOC concentration analysis in tumors.

### *In vitro* and *in vivo* therapy

### *In vitro* therapy

**Figure 5:**
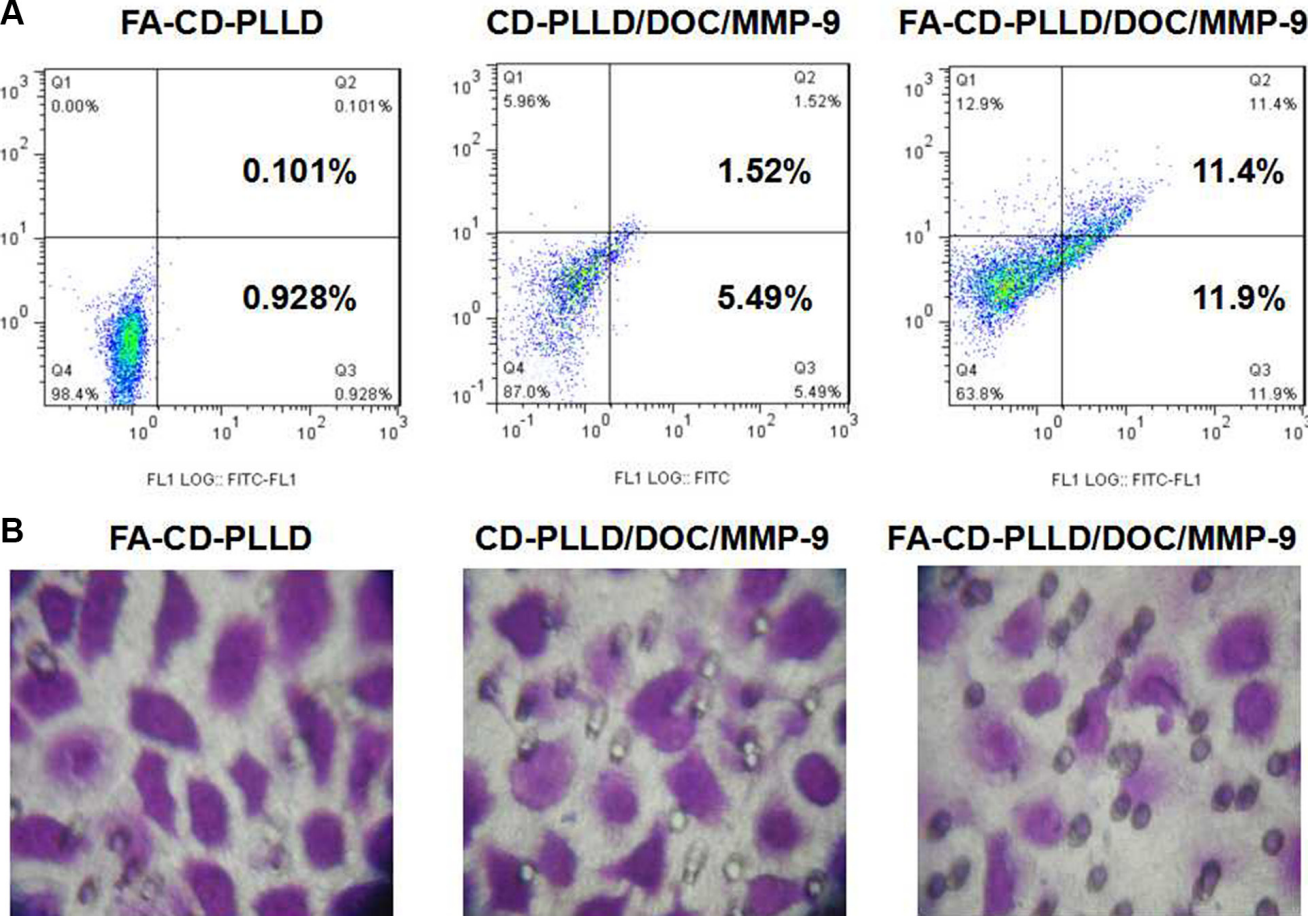
Apoptosis analysis (A) and invasion assay (B) on HNE-1 cells incubated with various samples

To explore whether the targeting co-delivered DOC and MMP-9 could induce HNE-1 cells apoptosis more effectively, the percentage of cell apoptosis treated with various formulations was determined by flow cytometer. As shown in Figure 5A, after incubation with FA-CD-PLLD for 48 h, HNE-1 cells displayed limited apoptosis and the apoptosis ratio showed no significant difference with the control group, which demonstrated the non-toxicity of FA-CD-PLLD. After loading DOC and MMP-9, the complexes showed obvious apoptosis to HNE-1 cells. It was found that for the cells treated with CD-PLLD/DOC/MMP-9, about 7% HNE-1 cells undergone apoptosis. Further more, the percentage of HNE-1 apoptosis treated with FA-CD-PLLD/DOC/MMP-9 reached up to 23.3%, much higher than that treated with non-targeting CD-PLLD/DOC/MMP-9. These results revealed that targeting co-deliverer of both MMP-9 and DOC can significantly enhance the cell apoptosis

To determine whether the targeting delivery of both DOC and MMP-9 renders HNE-1 cells less invasion, a transwell invasion assay was conducted on blank FA-CD-PLLD, CD-PLLD/DOC/MMP-9 and FA-CD-PLLD/DOC/MMP-9. From Figure 5B, it was found that FA-CD-PLLD/DOC/MMP-9 significantly decreased cells invasion than the group of CD-PLLD/DOC/MMP-9, and the ratio of HNE-1 cells which were able to invade through matrigel decreased obviously. FA-CD-PLLD/DOC/MMP-9 showed the better therapy effect to HNE-1 cells *in vitro* compared with non-targeting CD-PLLD/DOC/MMP-9 through flow apoptosis and cell invasion assays, which benefited from the molecular targeting of FA.

### *In vivo* therapy

**Figure 6:**
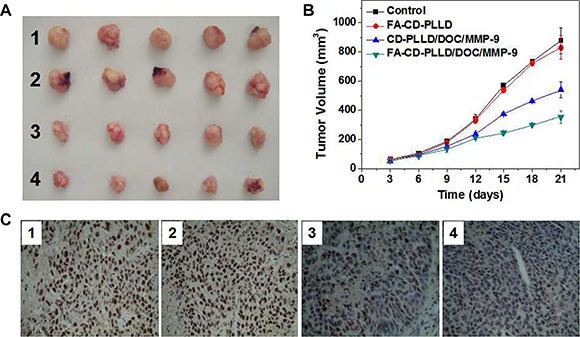
(**A**) A representative image of the HNE-1 tumor at the 21th day after treatment with various formulations. (**B**) Anti-tumor effects after various treatment on mice (*n* = 5). (**C**) PCNA expression in HNE-1 tumor. (1: control; 2: FA-CD-PLLD; 3: CD-PLLD/DOC/MMP-9; 4: FA-CD-PLLD/DOC/MMP-9).

The anti-tumor effect *in vivo* of FA-CD-PLLD/DOC/MMP-9 was tested, and PBS was used as the control. Nude mice implanted with HNE-1 tumors were used as the animal models for this *in vivo* anti-tumor test. The representative tumor images are shown in Figure 6A. As is seen, the tumor volume of the FA-CD-PLLD treated group increased rapidly after 21 d, and had no significant difference in volume with the PBS control. However, both of the DOC/MMP-9 treated groups were effective in tumor regression, and more obvious inhibition effect on HNE-1 tumor was found when treated with FA-CD-PLLD/DOC/MMP-9. Figure 6B gave the HNE-1 tumor growth profiles after treated with different groups, and the same results were obtained. The group treated with FA-CD-PLLD/DOC/MMP-9 showed the smallest volume and slowest growth rate.

PCNA is a cofactor of DNA polymerase δ which is necessary for cell proliferation, so it is often used as an index of DNA replication and cell proliferation [23]. Tumor PCNA expression in each group were shown in Figure 6C. It was found that high level expression of PCNA was observed in normal control group with the PI of 94.02%, and the PI of the blank FA-CD-PLLD group, CD-PLLD/DOC/MMP-9 group and FA-CD-PLLD/DOC/MMP-9 group were 90.53%, 48.73% and 5.16% respectively. Compared with the control group and blank FA-CD-PLLD group, PI in both CD-PLLD/DOC/MMP-9 group and FA-CD-PLLD/DOC/MMP-9 group were decreased obviously, and FA-CD-PLLD/DOC/MMP-9 group showed the best result.

### *In vivo* DOC and MMP-9 distribution

**Figure 7:**
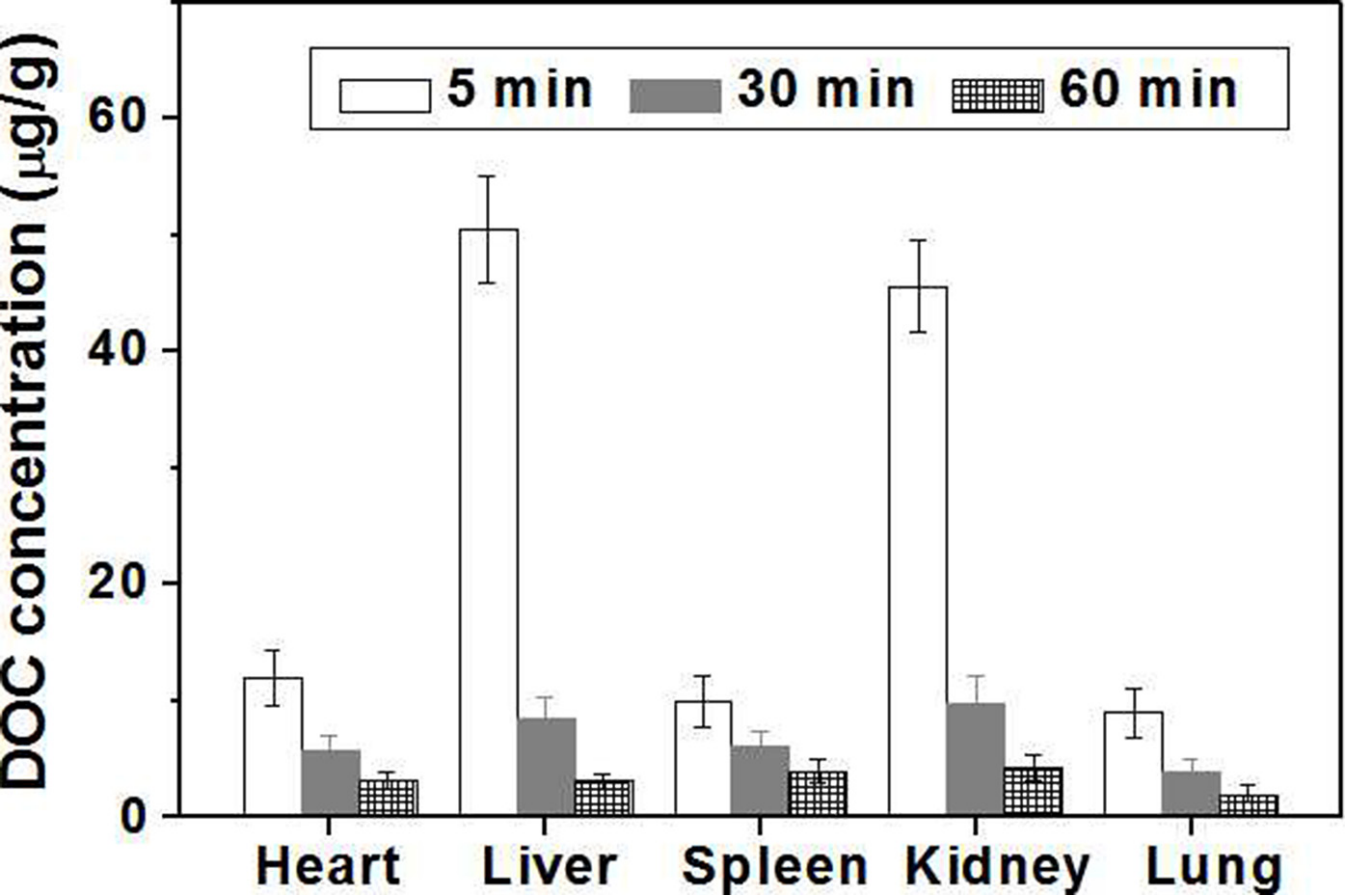
DOC concentrations in main organ of mice at different time after treatment with FA-CD-PLLD/DOC/MMP-9 (*n* = 5)

DOC concentration in each tissue (liver, kidney, lung, heart, spleen and brain) was assayed according to tert butyl methyl ether extraction method [24], and the standard curves and regression equations were achieved and shown in Electronic Supplementary Information. The mean tissue concentrations of DOC after intravenous administration of the FA-CD-PLLD/DOC/MMP-9 at the DOC dose of 20 mg/kg at different times are shown in Figure 7. The results demonstrated that DOC was absorbed rapidly and distributed widely into most tissues after intravenous administration of FA-CD-PLLD/DOC/MMP-9, and the DOC concentration was the highest in liver, followed by kidney, heart, spleen and lung. After 60 min circulation, DOC concentration decreased obviously and there was no significant difference in different organs. In addition, no DOC was detected in the brain of the nude mice.

**Figure 8:**
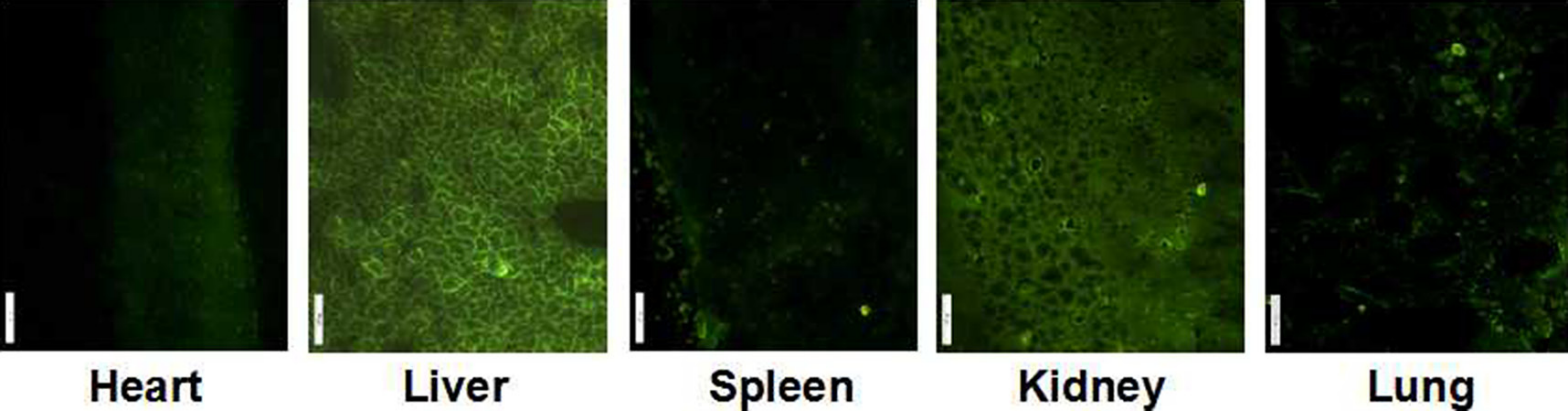
GFP expression in main organ of mice after 24 h treatment with FA-CD-PLLD/DOC/MMP-9

The MMP-9 distribution was also studied through GFP expression observed in tissue frozen section, shown in Figure 8. Similar to DOC distribution, GFP expression can be observed in all assayed organs except brain, and liver and kidney frozen sections showed stronger GFP.

### Biocompatibility

**Figure 9:**
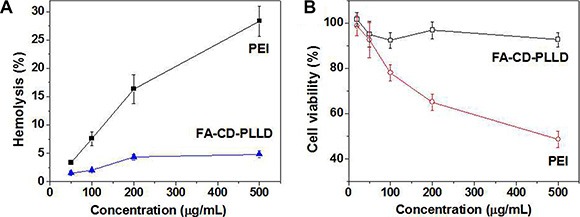
(**A**) Effect of the FA-CD-PLLD concentrations and PEI on hemolysis (*n* = 5). (**B**) MTT results of FA-CD-PLLD and PEI at different concentrations on HEN-1 cells (*n* = 5).

The biocompatibility is a key factor for FA-CD-PLLD as the drug and gene dual carrier. Herein, the hemolysis, *in vitro* and *in vivo* toxicity were tested to confirm the good biocompatibility of FA-CD-PLLD. The blood compatibility of FA-CD-PLLD was assessed by spectrophotometric measurement of hemoglobin release from erythrocytes after polymer treatment. Figure 9A showed the percentage of blood in contact with FA-CD-PLLD or PEI with different concentrations. It was found that PEI caused serious hemolysis in a concentration-dependent manner as a result of the erythrocyte membrane disruption, while FA-CD-PLLD showed a much better blood compatibility. When the FA-CD-PLLD concentration was up to 500 μg/mL, it showed non-hemolytic with the extent of hemolysis lower than the permissible level of 5% [25].

The cytotoxicity of FA-CD-PLLD was evaluated on HNE-1 cells by an MTT assay. Figure 9B gave the cell viabilities result of the HNE-1 cells cultured in the media treated with different FA-CD-PLLD or PEI concentrations. As seen, FA-CD-PLLD had an obviously lower toxicity than PEI. The cell viability was higher than 90% even if FA-CD-PLLD concentration reached as high as 500 μg/mL. Contrarily, the viability of HNE-1 cells treated with 500 μg/mL PEI was lower than 50%.

**Figure 10:**
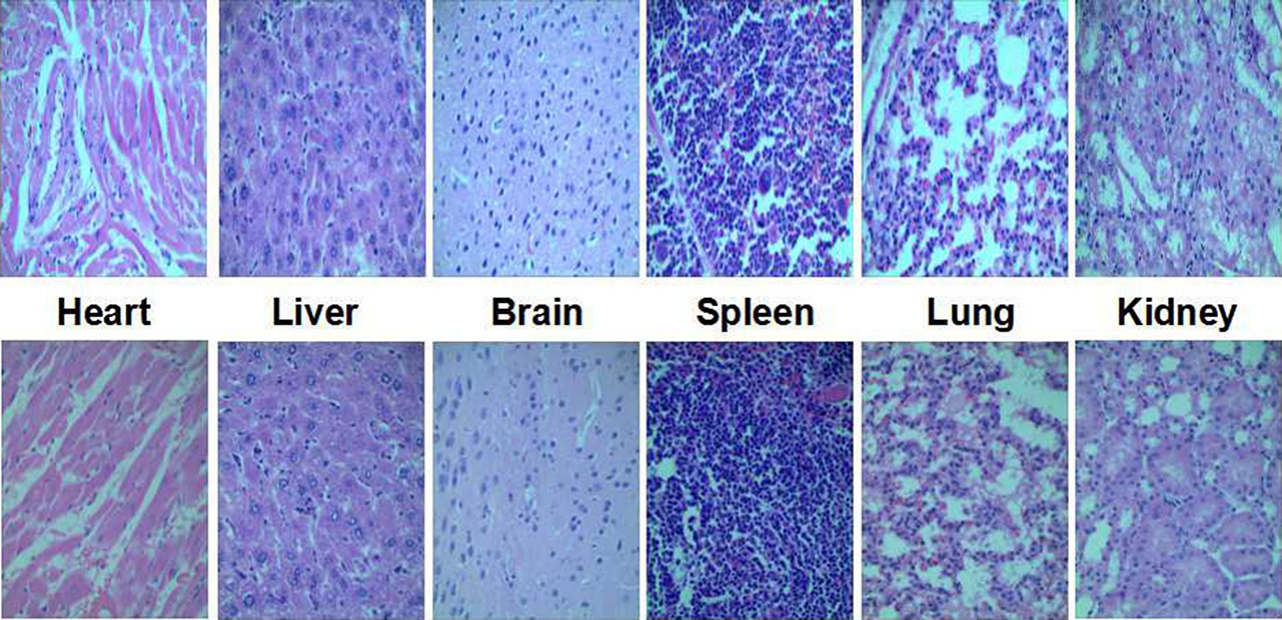
Representative organ histology for control (top row) and FA-CD-PLLD (bottom row) injected mice

*In vivo* toxicity studies are essential to prove the safety of any polymers used as gene delivery. Herin, a histological analysis of organs was performed to determine whether FA-CD-PLLD caused tissue damage, inflammation, or lesions. As shown in Figure 10, histologically, no visible difference was observed compared to the control (top row).

## DISCUSSION

NPC is a common malignant tumor in many countries, especially in South China. Nowadays, treatment of radiotherapy combined with chemotherapy is the most common clinical strategy, but due to the tumor metastasis and multi-drug resistance and so on, the overall 5 years survival rate is about 50%. The co-delivery of drug and gene has become the primary strategy in cancer and other disease therapy in recent years [3].

Our collaborators have synthesized a star-shaped cyclodextrin derivative (CD-PLLD) consisting of a cyclodextrin (CD) core and poly(L-lysine) dendron (PLLD) arms [10]. We have used this CD-PLLD to co-deliver docetaxel and MMP-9 siRNA plasmid for NPC therapy [15]. However, the deliver efficiency of CD-PLLD should be improved. Using folate as the ligand, a wide variety of drug payloads can be delivered to FR-positiver cells, ranging from small radioactive imaging agents up to large DNA-containing formulations, folate modified nanomedicine had more obvious inhibition effect on tumor cells in a short treating time and lower concentration via molecular targeting phagocytosis FR pathway [26, 27]. In this paper, we conjugated CD-PLLD with FA by the amidation reaction to synthesize a FA-targeted drug and gene dual carrier, and then use this carrier to co-deliver DOC and MMP-9 for NPC treatment.

From *in vitro* target results, it was confirmed that the FA-CD-PLLD could targeting deliver MMP-9 siRNA plasmid effectively into HNE-1 cells. *In vivo* target experiment, for DOC distribution analysis, compared to liquid–liquid anhydrous diethyl ether extraction method [28], we found tissue sample extracted by low toxicity tert-butyl methyl ether can be blown dry easily by nitrogen, and the higher DOC extraction rate in tissue was acquired [24]. Because of the two compartment model of drug distribution, after tail intravenous injection, nanomedicines were distributed immediately in central compartment of organs with abundant blood supply such as liver, heart, kidney and so on, concentration of DOC dropped fastly following with accelerated blood flow, and then nanomedicines were distributed slowly in peripheral compartment of organs with less blood flow such as skin, fat, bone and so on, and GFP expression *in vivo* was similar to DOC distribution. These results indicated that FA-CD-PLLD/DOC/MMP-9 complexes could be stable in blood circulation and could not escape phagocytosis effectively in meshy endodermis system such as liver and spleen, which need to improve further. Moreover, the complexes could not cross the blood–brain barrier because of its larger molecular size which confirmed its relative safety. Because the nanomedicine can be swallowed by tumor cell easily and accumulate in the tumor tissues for its enhanced permeability and retention effect (ERP effect) [29, 30], so DOC concentrations in both HNE-1 and CNE-2 tumor increased with the prolonged blood circulation time. Moreover, the DOC concentration in HNE-1 tumor was significantly higher than that in CNE-2 tumor because of the molecular targeting property of FA-CD-PLLD/DOC/MMP-9 nanocomposite. GFP expression in HNE-1 tumor was stronger than that in CNE-2 tumor, which was consistent with the DOC concentration analysis in tumors. It showed FA-CD-PLLD/DOC/MMP-9 nanocomposite had a good targeting ability to achieve the high DOC concentration and gene transfection efficiency in HNE-1 tumor. and FA-CD-PLLD was considered as a promising targeting gene and drug carrier.

Cell apoptosis was determined by flow cytometer. Annexin V-FITC staining in conjunction with PI can distinguish early apoptosis from late apoptosis or living cells from necrotic cells [31]. *In vitro* therapeutic results revealed FA-CD-PLLD/DOC/MMP-9 showed the better therapeutic effect on HNE-1 cells compared to non-targeting CD-PLLD/DOC/MMP-9 through flow apoptosis and cell invasion assays, which benefited from the molecular targeting of FA. *In vivo* treatment results also showed anti-tumor effcet was most obvious in FA-CD-PLLD/DOC/MMP-9 treated group by analysed tumor volume and PCNA expression. The results were attributed to the targeting effect of FA-CD-PLLD, which could accumulate DOC and MMP-9 in tumor tissue and penetrate into tumor deep [32]. The targeting co-delivering strategy is promising in cancer therapy [32].

The instability of delivery vehicles in the blood was considered as one of the serious limitations in the therapeutic of cationic polymers [33]. The nonspecific interactions of cationic polymers with blood components could severely diminish the half-life and targetability of complexes. FA-CD-PLLD showed a much better blood compatibility and *in vitro* obviously lower toxicity than PEI. The *in vivo* toxicity of polymers is influenced by the chemical structures, size, exposure duration, biodistribution, location, metabolism as well as the nature of the surface and terminal groups. The toxicity of FA-CD-PLLD also depends on its type, molecular weight and generation [34]. The non-observed toxicity of star-shaped FA-CD-PLLD could be attributed to its lower molecular weight (less than 5 KDa) and the characteristic of molecular structure, such as the biodegradability and carrying a positive charge only on the surface. The biodegradability of FA-CD-PLLD can also promote its elimination from organism and thereby enhance the *in vivo* biocompatibility [35].

In conclusion, *in vitro* and *in vivo* assays showed that FA-CD-PLLD/DOC/MMP-9 as the co-delivery of hydrophobic drug and gene can efficiently exert targeted anti-tumor effect. It indicated a promising strategy for targeted NPC synergy therapy.

## MATERIALS AND METHODS

### Materials

CD-PLLD has been synthesized according to our previous work [10]. *Doc* etaxel (DOC) was purchased from Sigma and used without further purification. MMP-9 siRNA plasmid vector expressing EGFP was purchased from Invitrogen Company (Shanghai). 1-hydroxybenzotriazole (HOBt), O-benzotriazole-N,N,N′,N′-tetramethyluromium hexafluorophosphate (HBTU) and folate (FA) were obtained from Aladdin Ltd (Shanghai) and dried before use. RPMI-1640 medium, fetal bovine serum, Propidium Iodide (PI) and PBS purchased from Invitrogen Company. Human NPC HNE-1 cells and CNE-2 cells were provided by Southern Medical University. BALB/c mice and BALB/c nude mice (SPF) bought from laboratory animal center, Southern Medical University. The Institutional Administration Panel for Laboratory Animal Care approved the experimental design. The university guidelines for care and use of laboratory animals were strictly followed.

### Synthesis of FA-CD-PLLD

The dried CD-PLLD (0.76 g, 0.1 mmol) and FA (0.132 mg, 0.3 mmol) were dissolved in 20 mL dried DMF, then 0.3 mmol HOBT and 0.3 mmol HBTU were added. After 48 h reaction at room temperature, the mixture was dialyzed in distilled water for 3 d (MWCO = 3000). The FA-CD-PLLD was obtained by lyophilization with a yield of 68%. Its chemical structure was characterized by ^l^H NMR analyses. The ^1^H NMR spectra were measured in DMSO-*d*_6_ by using a Bruker DPX-300 NMR spectrometer (300 MHz) at 25°C.

### DOC loading

For the loading of hydrophobic DOC, 50 mg FA-CD-PLLD was dissolved in distilled water with a concentration of 10 mg/mL, then 5 mL DMF containing 15 mg DOC was added dropwise to the solution. The mixture was stirred for 4 h in the dark at room temperature. After that, the sample was put into a dialysis bag (MWCO = 500) and subjected to dialysis against distilled water for 24 h. The drug-loaded complex was obtained by filtered through a 0.45 μm filter and then lyophilized.

To determine the loading amount of DOC, the resultant FA-CD-PLLD/DOC complexes were dissolved in CH_3_OH and then analyzed by HPLC. The HPLC analysis of DOC was achieved on a C_18_ column (Waters, USA) with a mobile phase consisting of methanol and purity water (70/30, v/v) at a flow rate of 1.0 mL/min. The effluents were monitored at 227 nm and quantized by comparing the peak areas with the standard curve [36]. It was found that the loading amount of DOC in FA-CD-PLLD was 38.4 μg/mg.

### MMP-9 binding

The best siRNA plasmid targeting MMP-9 has been screened out in our previous work [15], showing in Electronic Supplementary Information. For FA-CD-PLLD, the optimized N/P ratio was 20, which was shown in Electronic Supplementary Information (Supplementary Figure S1). Then, FA-CD-PLLD and MMP-9 were dissolved in distilled water to make aqueous solutions respectively and then mixed at an N/P ratio of 20. The resultant mixture was stirred gently for 15 min for the formation of FA-CD-PLLD/MMP-9 complexes. For the preparation of co-loaded nanoparticles, FA-CD-PLLD/DOC complex was mixed with MMP-9 at an N/P ratio of 20. The resultant mixture was stirred gently for 15 min for the formation of FA-CD-PLLD/DOC/MMP-9 nanoparticles.

### Targeting analysis

### *In vitro* transfection

HNE-1 and CNE-2 cells were selected for studying the *in vitro* gene transfection of the targeted FA-CD-PLLD/MMP-9 complexes. Before transfection, HNE-1 and CNE-2 cells were seeded at a density of 1 × 10^4^ cells per well onto 12-well tissue culture plates in complete DMEM (Dulbecco's Modified Eagle Medium) culture medium respectively, and then incubated in a humidified 5% CO_2_ atmosphere at 37°C. Freshly prepared FA-CD-PLLD/MMP-9 (N/P = 20) complexes in serum-free RPMI-1640 were added. The MMP-9 in each well was fixed at 3.0 μg. After 2 h incubation, the formulations were removed and 500 μL of fresh DMEM culture medium was added. The experiments of studying gene transfection were continued to 46 h, and then the cells were analyzed for green fluorescence protein (GFP) expression with a fluorescence microscope (Nikon-2000 U, Japan). The cells treated with PEI/MMP-9 (N/P = 10) were set as the control groups. After the cells were digested by trypsinase (0.05 wt% in PBS), the transfection percents (positive cell percent) were calculated by dividing the number of fluorescent cells by the number of total cells in a certain area of a well. The transfection efficiency was recorded by a flow cytometer (Accuri C6).

### mRNA and protein expression

HNE-1 cells (5 × 10^4^) were seeded in 6-well plates and incubated at 37°C in 5% CO_2_ for 24 h to reach 70% confluence. Various formulations (Blank FA-CD-PLLD, CD-PLLD/MMP-9 and FA-CD-PLLD/MMP-9) were added and incubated with the cells for 48 h (for mRNA isolation and protein extraction). The cellular levels of MMP-9 mRNA and protein were assessed using RT-PCR and Western blot, respectively.

In RT-PCR analysis, total RNA from transfected cells was isolated using the AxyPrep Multisource total RNA Miniprep Kit (Axygen, USA) according to the protocol of manufacturer. 1 mg of total RNA was transcribed into cDNA using the PrimeScript^TM^ RT reagent Kit with gDNA Eraser (Takara, Japan). Thereafter, 2 μL of cDNA was subjected to RT-PCR analysis targeting MMP-9 and β-actin using Premix Taq Version 2.0 (Takara, Japan). PCR parameters consisted of 35 cycles of PCR (denaturation at 95°C for 30 sec, annealing at 58°C for 30 sec, and elongation at 72°C for 30 sec). The PCR products were run on 2% agarose gel with Ethidium bromide and visualized in Gel Doc^TM^ XR+ imaging system (Bio-Rad). Relative gene expression values were determined using Quality One Software. Primers used in RT-PCR for MMP-9 and β-actin are:

MMP-9-forward 5′-GAGAAGAGAGGGCCCAGC-3′

MMP-9-reverse 5′-ACGTGACCTATGACATCCT GC-3′ and

β-actin-forward 5′-CGGGAAATCGTGCGTGAC-3′

β-actin-reverse 5′-TGGAAGGTGGACAGCGAGG-3′

In Western blot analysis, transfected cells were washed twice with cold PBS, and then resuspended in 100 μL of lysis buffer (50 mM Tris-HCl, pH = 7.4, 150 mM NaCl, 1% Triton X-100, 10% glycerol, 1.5 mM MgCl_2_, 1 mM EDTA) freshly supplemented with Roche's Protease Inhibitor PMSF Tablets. The cell lysates were incubated on ice for 30 min and vortexed every 5 min. The lysates were then clarified by centrifugation for 10 min at 12 000 r/min. The supernatant was boiled in loading buffer for 10 min. Total protein (20 μL) was separated (at 120 V for 40 min) on 12% PAGE-SDS gels and then transferred (at 300 mA for 40 min) to PVDF membranes (Bio-Rad). After incubation in 5% BSA (Merck, Germany) in phosphate buffered saline with Tween-20 (PBST, pH 7.2) for 1 h. The membranes were incubated in 5% BSA in PBST with MMP-9 antibodies (1:1000) over night. After incubation in 5% BSA in PBST with goat anti rabbit IgG-HRP antibody (1:5000) for 60 min, bands were visualized using the ECL system (Pierce). Relative gene expression values were determined using Image-J Software.

### *In vivo* assay

Nude mice implanted with HNE-1 and CNE-2 tumors were used as the animal model for *in vivo* targeting analysis. HNE-1 cells (1 × 10^7^ cells in 200 μL PBS) and CNE-2 cells (1 × 10^7^ cells in 200 μL PBS) were injected subcutaneously on the right and left axillary flank of female BALB/c nude mice respectively. After the tumors grown with suitable size a few days later, the nude mice were sacrificed, and the removed HNE-1 and CNE-2 tumors were cut into small patchs at aseptic conditions. Then, the tumors were transplanted to the right and left axillary flank of 5 nude mice respectively, and all nude mice were aged 4–5 weeks and weighed 17–20 g. Then all nude mice were anesthetized by intraperitoneal injection of chloral hydrate, and injected with FA-CD-PLLD/DOC/MMP-9 through tail vein at DOC doses of 19.2 μg/g and MMP-9 doses of 25 μg/g.

### DOC distribution

After administration, nude mice bearing HNE-1 and CNE-2 tumor were sacrificed at 5 min, 30 min and 60 min respectively, and the tumors were rapidly resected. Tumor samples were rinsed with ice saline, dried by filter paper and homogenized with saline according to 1:2 ratio of tumor weight/saline doses. After that, the tumor samples were extracted by tert-butyl methyl ether. 0.2 mL of tumor tissue sample and 1.2 mL of tert-butyl methyl ether were mixed for 5 min by vortex to extract, then the total organic layer was separated by centrifugation at 10000 rpm for 10 min, transferred to a clean tube, and evaporated to dryness at 40°C under a stream of nitrogen. The drug residue was finally reconstituted in 0.4 mL acetonitrile followed by centrifugation at 10000 rpm for 5 min before analysis. The DOC concentrations were determined by the HPLC.

### GFP expression

Nude mice were sacrified after 24 h via tail vein injection of nanocomposites, HNE-1 tumor and CNE-2 tumor were *peeled off* immediately, and GFP expression in the tumor tissue frozen section was observed under the fluorescence microscope.

### NPC HNE-1 therapy

### Apoptosis assay

HNE-1 cells seeded on the 24-well plates were treated with FA-CD-PLLD, CD-PLLD/DOC/MMP-9 and FA-CD-PLLD/DOC/MMP-9 (DOC concentration of 0.192 μg and MMP-9 concentration of 2 μg/well) at 37°C for 48 h. Cells without treatment were used as control. At the end of incubation, all cells were trypsinized, collected and resuspended in 200 μL of binding buffer. Thereafter, 5 μL of annexin V-FITC and 10 μL of PI were added and mixed for 15 min in the dark. The stained cells were analyzed using a flow cytometer.

### Cell invasion assay

For invasion assays, the cells were plated into 6-well dishes in triplicate at high density. The cells were serum starved for 12 h before performing the assay, and BD BioCoat Matrigel Invasion Chambers were used for invasion assays. After starvation, cells were trypsinized and 2.5 × 10^4^ cells were plated in 0.2 mL PRMI 1640 with 0.5% FBS in the upper chamber. In the lower chamber, 0.5 mL PRMI 1640 with 10% FBS was used as an attractant. Cells were incubated for 24 h at 37°C. Invading cells on the lower surface of the membranes were stained with 0.3% crystal violet stain and counted manually.

### *In vivo* tumor inhibition

Nude mice implanted with HNE-1 tumors were used as the animal model for *in vivo* anti-tumor test. The mice were divided into 4 groups randomized, each group was 5 mice. Subsequently, the mice were intravenously injected via tail vein with 200 μL PBS (pH=7.4, as control), FA-CD-PLLD, CD-PLLD/DOC/MMP-9 and FA-CD-PLLD/DOC/MMP-9 at DOC doses of 9.6 μg/g and MMP-9 doses of 12.5 μg/g at the 1st, 6th, 11th and 16th day respectively. After 3 weeks, all groups of mice were sacrificed. The tumor volume was calculated by the formula W × L^2^/2, where W is the widest diameter, and L is the longest diameter.

The tumor tissues from the mice treated with different samples were selected for histology observation on the 21st day after treatment. The tumors were dissected and fixed in 10% neutral buffered formalin, routinely processed into paraffin, sectioned at a thickness of 5 mm. The proliferating cell nuclear antigen (PCNA) was used to evaluate the proliferation ability of the cells in the left tumors. The slices obtained were examined by optical microscopy.

### *In vivo* DOC and MMP-9 distribution

For DOC and MMP-9 *in vivo* distribution, the nude mice bearing HNE-1 tumor model were firstly established described as 2.5.3. Then the nude mice were intravenously injected via tail vein with FA-CD-PLLD/DOC/MMP-9 at DOC doses of 19.2 μg/g and MMP-9 doses of 25 μg/g.

### DOC distribution

After administration, nude mice were sacrificed at 5 min, 30 min and 60 min respectively. The main tissues (liver, kidney, lung, heart, spleen and brain) were rapidly resected respectively. Tissue samples were removed, rinsed with ice saline, dried by filter paper and homogenized with saline according to 1:2 ratio of tissue weight/saline doses respectively. The DOC concentrations were determined by the HPLC.

### MMP-9 distribution

Nude mice were sacrified after 24 h via tail vein injection of nanocomposites, each tissue (liver, kidney, lung, heart, spleen and brain) was resected immediately, and GFP expression in the tissue frozen section was observed under the fluorescence microscope.

### Biocompatibility

### Blood compatibility

The blood compatibility of CD-PLLD was evaluated by its hemolysis assay. For each sample, its hemolytic potential was tested according to the method reported by O'Leary and Guess [37]. Human blood (0.1 mL) anticoagulated with citrate was added to 5 mL of PBS containing the samples with different amounts in test tubes. Separate positive (100% hemolysis induced by replacing the PBS with 5 mL of 0.1% Na_2_CO_3_ solution) and negative (0% hemolysis, PBS with no material added) controls were also set up. Each set of experiments was carried out for three times. All the test tubes containing the samples and the control were incubated for 1 h at 37°C. After the incubation, the tubes were centrifuged at 500 rpm for 5 min. The percentage hemolysis was calculated by measuring the optical density (OD) of the supernatant solution at 545 nm in a UV-Vis spectrophotometer as per the following formula:

Hemolysis (%) = [(OD of the test sample - OD of negative control) × 100]/OD of positive control.

### Cell viability

HNE-1 cells were cultured onto a 96-well plate (1 × 10^4^ cells/well) in complete DMEM (with high glucose and 10% fetal bovine serum supplemented) in a humidified atmosphere of 5% CO_2_ at 37°C. After 24 h, the growth medium was replaced with 200 μL complete DMEM culture medium that contained the desired amount of FA-CD-PLLD or PEI respectively. Five multiple holes were set for every sample. The cells treated with the same amount of PBS were used as a control group. The cells were incubated for another 48 h, and the cell viability was assayed by adding 20 μL of MTT (Sigma) PBS solution (5 mg/mL). After incubation at 37°C for another 4 h, the formed crystals were dissolved in 150 μL of DMSO. The absorbance that correlated with the number of viable cells in each well was measured by an MRX-Microplate Reader at a test wavelength of 490 nm.

### *In vivo* toxicity

The FA-CD-PLLD (500 mg/kg mouse) was dissolved in PBS and injected into 7 female BALB/c mice (4-week old, 18 ± 2 g) through tail vein, and physiological saline was used as control reagent. After 7 days, all animals were sacrificed, and the liver, heart, brain, spleen and kidney were separated, washed twice with PBS and fixed in 4% formaldehyde for histological examination.

### Statistical analysis

Comparison between groups was analyzed by the one-tailed Student's *t*-test using statistical software SPSS 11.5. All data are presented as means ± S.D. Differences were considered to be statistically significant when the *P* values were less than 0.05.

## CONCLUSIONS

For the targeting co-delivery of hydrophobic drug and gene effectively, a FA modified star-shaped copolymer (FA-CD-PLLD) has been synthesized and used to targeting co-deliver DOC and MMP-9 for nasopharyngeal cancer therapy. The obtained FA-CD-PLLD showed a good molecular targeting ability *in vitro* and *in vivo*, and could targeting mediate a significant reduce of MMP-9 protein in HNE-1 cells. The FA-CD-PLLD/DOC/MMP-9 could enhance the DOC/MMP-9 delivery effect obviously, inducing a more significant apoptosis and decreasing invasive capacity of HEN-1 cells. *In vivo* assays showed that FA-CD-PLLD/DOC/MMP-9 could inhibit HNE-1 tumor growth and decrease PCNA expression effectively. Moreover, FA-CD-PLLD showed the good blood compatibility and non-toxicity, indicating a promising strategy for nasopharyngeal carcinoma therapy.

## SUPPLEMENTARY FIGURES


