# Supplementary material for: Folate-targeted star-shaped cationic copolymer co-delivering docetaxel and MMP-9 siRNA for nasopharyngeal carcinoma therapy
Source: Oncotarget. 2016 Jun 1;7(27):42017–30. doi: 10.18632/oncotarget.9771 (PMC5173113; doi:10.18632/oncotarget.9771)
Supplement: Supplementary file 1 [file oncotarget-07-42017-s001.pdf]

Folate-targeted star-shaped cationic copolymer co-delivering docetaxel and MMP-9 siRNA for nasopharyngeal carcinoma therapy

Supplementary Materials

MMP-9 siRNA screening and N/P ratios optimization

The best siRNA sequences targeting MMP-9 was following

MMP-9 siRNA-forward 5′-TGCTGAAACCGA GTTGGAACCAC GACGTTTTGGCCACTGACTGACG TCGTGG TCAACTCGGTTT-3′; MMP-9 siRNA-reverse 5′-CCTGAAACCGAGTTGACCACGACGTC AGTCAG TGGCCA AAACGTCGTGGTTCCAACTCGGTTTC-3′

Transfection optimization

The transfection eficiency of FA-CD-PLLD/ MMP-9 complexes at different N/P ratios (10, 20 and 40 respectively) were evaluated by *in vitro* gene transfection assay using HNE-1 cells. The PEI/MMP-9 complex was also investigated as the control group at an N/P ratio of 10, at which PEI could achieve the highest level of transfection efficiency [1]. Supplementary Figure S1 showed the transfection results of the transfected HNE-1 cells in these cases. It was found that PEI showed a high transfection efficiency and about 49.0% HNE-1 cells were transfected. Although FA-CD-PLLD showed a reduced transfection efficiency compared with PEI, it also had the receivable gene transfection ability at an N/P ratio of 20, at which more than 25% HNE-1 cells were transfected. Moreover, the transfected cells at the N/P ratio of 40 were slightly less than those at the N/P ratio of 20. This result may be caused by the slightly reduced tolerance of tumor cells under the high FA-CD-PLLD concentration [2].

Standard curves of DOC concentration for *in vivo* distribution

Methods

Firstly, standard curves of DOC concentration in each tissue (liver, kidney, lung, heart, spleen and brain) were achieved by the HPLC analysis. Briefly, 12.16 mg DOC standard samples were transferred into a 5 mL calibrated flask, solved with the mobile phase solvent to 5 mL. Then the DOC solution was diluted with mobile phase solvent to the following gradient DOC concentration: 1216, 608, 304, 152 and 76 µg/mL.

Nude mice bearing HNE-1 tumor model were established and each tissue (liver, kidney, lung, heart, spleen and brain) was removed respectively. The tissues were rinsed with ice saline, dried by filter paper, weighed and homogenized with saline (1 g/2 mL ratio of tissue weight/saline doses).

Then, 160 µL of tissue sample and 40 µL of prepared DOC standards sample solution with gradient concentration were mixed, and 1 mL of tert-butyl methyl ether was added into each sample and mixed for 5 min by vortex to extract. The total organic layer was separated by centrifugation at 10000 rpm for 10 min, transferred to a clean tube and evaporated to dryness at 40°C under a stream of nitrogen. The drug residue was finally reconstituted in 0.4 mL acetonitrile followed by centrifugation at 10000 rpm for 5 min before analysis, of which 10 µL supernatant fluid were injected into the HPLC system.

RESULTS

The standard curve and regression equation were achieved as

liver: $Y = 2693.0 \times - 846.12$,
kidney: $Y = 2655.5 \times + 1238.8$,
lung: $Y = 2581.3 \times - 172.32$,
heart: $Y = 2608.7 \times - 701.39$,
spleen: $Y = 2470.4 \times + 266.92$,
brain: $Y = 2755.0 \times + 1459.2$.

REFERENCES

1. Hu C, Zhang L, Wu D, Cheng S, Zhang X, Zhuo R. Heparin-modified PEI encapsulated in thermosensitive hydrogels for efficient gene delivery and expression. J Mater Chem 2009; 19:3189–97.
2. Ma D, Zhao Y, Zhou XY, Lin QM, Zhang Y, Lin JT, et al. Photoenhanced gene transfection by a star-shaped polymer consisting of a porphyrin core and poly(L-lysine) dendron arms. Macromol Biosci 2013; 13:1221–7.

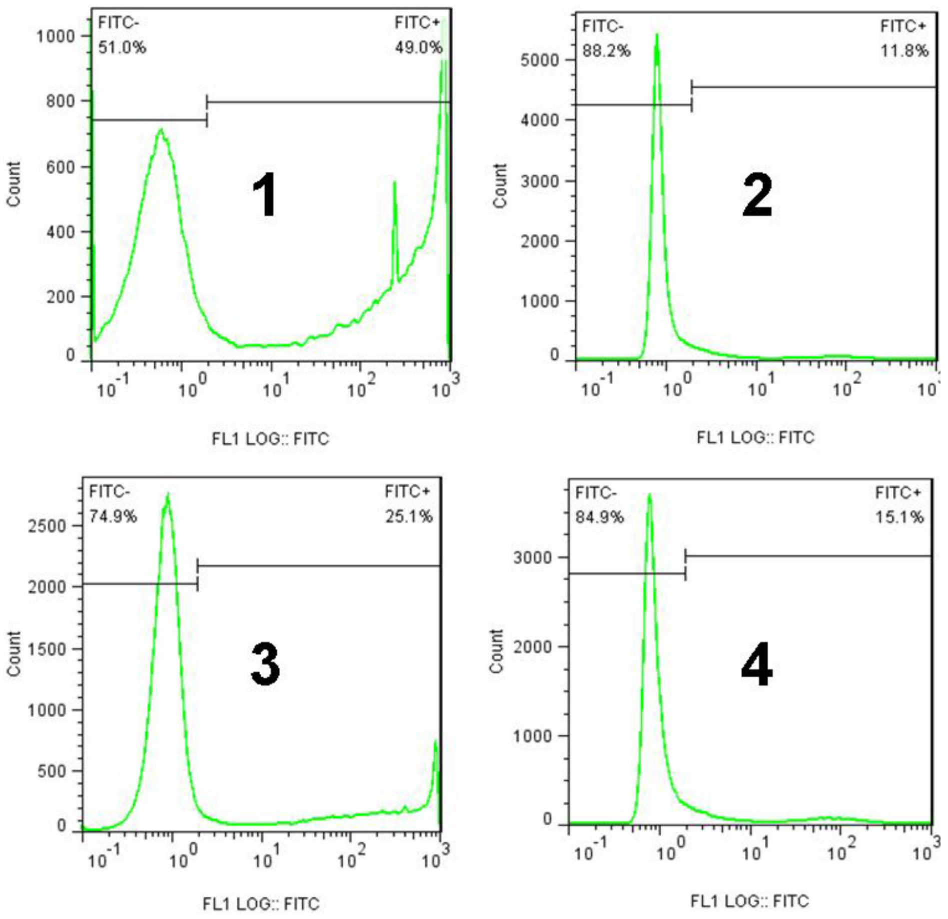

Supplementary Figure S1: The results of transfected HEN-1 cells determined by flow cytometry (1: PEI/MMP-9 with an N/P ratio of 10; 2-4: FA-CD-PLLD/MMP-9 with the N/P of 10, 20 and 40 respectively).
